# Supplementary material for: Structural damage detection and safety assessment method based on machine vision and machine learning
Source: PLoS One. 2026 Feb 2;21(2):e0341653. doi: 10.1371/journal.pone.0341653 (PMC12863553; doi:10.1371/journal.pone.0341653)
Supplement: Attachment — Submitted filename: Response to Reviewers.docx [file pone.0341653.s001.docx]

**Responses to the reviewers’ comments**

No.: PONE-D-25-17025

Title: Structural Damage Detection and Safety Assessment Method Based on Machine Vision and Machine Learning

Dear editor and reviewers:

Thank you very much for your comments and professional advice. These opinions help to improve academic rigor of our article. The authors have studied reviewer’s comments carefully and have made revision point-by-point response to the reviewer’s comments as provided below. Revisions implemented in accordance are also explained.

Our responses to the reviewers’ comments are given below, and the changed parts are highlighted with red in the revised manuscript. We sincerely hope the revised manuscript would be suitable for publication.

Thank you and best regards.

**Reviewer #1:** The manuscript needs some improvements before acceptance.

1. Highlight the novelty of the manuscript.

2. How does the integration of machine vision and machine learning enhance the efficiency and accuracy of damage detection in this proposed method?

3. What are the key factors driving the increasing reliance on infrastructure globally, as mentioned in the introduction?

4. The manuscript should include the recent publications. Authors should add the all below references:

i. Liu, H., Chen, J., Zhang, X., Dai, D., Cui, J.,... Spencer, B. F. (2024). Collaborative Imaging of Subsurface Cavities Using Ground-Pipeline Penetrating Radar. IEEE Geoscience and Remote Sensing Letters, 21, 1-5. doi: 10.1109/LGRS.2024.3390668

ii. Li, J., Hu, Z., Cui, J., & Lin, G. (2024). Efficient GPU-accelerated seismic analysis strategy and scenario simulation for large-scale nuclear structure cluster-soil interaction over ten million DOFs. Computers and Geotechnics, 174, 106583. doi: https://doi.org/10.1016/j.compgeo.2024.106583

iii. Tong, A., Zhang, J., & Xie, L. (2024). Intelligent Fault Diagnosis of Rolling Bearing Based on Gramian Angular Difference Field and Improved Dual Attention Residual Network. Sensors, 24(7), 2156. doi: https://doi.org/10.3390/s24072156

iv. Wang, H., Hou, Y., He, Y., Wen, C., Giron-Palomares, B., Duan, Y.,... Wang, Y. (2024). A Physical-Constrained Decomposition Method of Infrared Thermography: Pseudo Restored Heat Flux Approach Based on Ensemble Bayesian Variance Tensor Fraction. IEEE Transactions on Industrial Informatics, 20(3), 3413-3424. doi: 10.1109/TII.2023.3293863

v. Chen, H., Huang, B., Zhang, H., Xue, K., Sun, M.,... Wu, Z. (2024). An efficient Bayesian method with intrusive homotopy surrogate model for stochastic model updating. Computer-Aided Civil and Infrastructure Engineering, 39(16), 2500-2516. doi: https://doi.org/10.1111/mice.13206

vi. Zhang, C., Shu, J., Zhang, H., Ning, Y., & Yu, Y. (2024). Estimation of load-carrying capacity of cracked RC beams using 3D digital twin model integrated with point clouds and images. Engineering Structures, 310, 118126. doi: https://doi.org/10.1016/j.engstruct.2024.118126

vii. Wang, S., Lin, S., & Yang, R. (2024). A lightweight convolutional neural network for multipoint displacement measurements on bridge structures. Nonlinear Dynamics, 112(14), 11745-11763. doi: https://doi.org/10.1007/s11071-024-09673-x

viii. Hu, D., Hu, Y., Hu, R., Tan, Z., Ni, P., Chen, Y.,... Liu, J. (2024). Machine Learning–Finite Element Mesh Optimization-Based Modeling and Prediction of Excavation-Induced Shield Tunnel Ground Settlement. International Journal of Computational Methods, 2450066. doi: 10.1142/S021987622450066X

ix. Yin, Q., Xin, T., Zhenggang, H., & Minghua, H. (2023). Measurement and Analysis of Deformation of Underlying Tunnel Induced by Foundation Pit Excavation. Advances in Civil Engineering, 2023(1), 8897139. doi: https://doi.org/10.1155/2023/8897139

x. Li, D., Nie, J., Wang, H., Yu, T., & Kuang, K. S. C. (2025). Path planning and topology-aided acoustic emission damage localization in high-strength bolt connections of bridges. Engineering Structures, 332, 120103. doi: https://doi.org/10.1016/j.engstruct.2025.120103

xi. Li, D., Chen, Q., Wang, H., Shen, P., Li, Z.,... He, W. (2024). Deep learning-based acoustic emission data clustering for crack evaluation of welded joints in field bridges. Automation in Construction, 165, 105540. doi: https://doi.org/10.1016/j.autcon.2024.105540

xii. Xia, Z., Shu, J., Ding, W., Gao, Y., Duan, Y., Debono, C. J.,... Borg, R. P. (2025). Complete-coverage path planning for surface inspection of cable-stayed bridge tower based on building information models and climbing robots. Computer-Aided Civil and Infrastructure Engineering. doi: https://doi.org/10.1111/mice.13469

xiii. Li, Y., Weng, X., Hu, D., Tan, Z., Qi, K.,... Liu, J. (2025). Data-Driven Deep-Learning Model for Predicting Jacking Force of Rectangular Pipe Jacking Tunnel. Journal of Computing in Civil Engineering, 39(3), 4025017. doi: 10.1061/JCCEE5.CPENG-6167

xiv. Dai, N., Hu, X., Xu, K., Hu, X., Yuan, Y., Cao, B.,... Shi, L. (2025). Blind super-resolution network based on local fuzzy discriminative loss for fabric data augmentation. Journal of Engineered Fibers and Fabrics, 20(06). doi: 10.1177/15589250241313158

xv. Jiang, T., Tang, Y., Xu, C., & Liu, W. (2025). A Calibration and Error Evaluation Method of a Combined Tracking-Based Vision Measurement System for Meter-Scale Components. IEEE Transactions on Industrial Informatics, 1-10. doi: 10.1109/TII.2025.3547351

xvi. Zuo, C., Zhang, X., Zhao, G., & Yan, L. (2025). PCR: A Parallel Convolution Residual Network for Traffic Flow Prediction. IEEE Transactions on Emerging Topics in Computational Intelligence, 1-12. doi: 10.1109/TETCI.2025.3525656

xvii. Wang X, Guo S, Xu Z, Zhang Z, Sun Z, Xu Y. A Robotic Teleoperation System Enhanced by Augmented Reality for Natural Human–Robot Interaction.Cyborg Bionic Syst. 2024;5:Article 0098. https://doi.org/10.34133/cbsystems.0098

xviii. Wang, Y., Han, Z., Xu, X., & Luo, Y. (2024). Topology optimization of active tensegrity structures. Computers & Structures, 305, 107513. doi: https://doi.org/10.1016/j.compstruc.2024.107513

xix. Jia, Y., Chen, G., & Zhao, L. (2024). Defect detection of photovoltaic modules based on improved VarifocalNet. Scientific Reports, 14(1), 15170. doi: 10.1038/s41598-024-66234-3

xx. Lin, L., Ma, X., Chen, C., Xu, J., & Huang, N. (2024). Imbalanced Industrial Load Identification Based on Optimized CatBoost with Entropy Features. Journal of Electrical Engineering & Technology, 19(8), 4817-4832. doi: https://doi.org/10.1007/s42835-024-01933-5

xxi. Yuan, K., Lang, X., Cao, J., & Zhang, H. (2025). Model of oil pipeline tiny defects detection based on DDPM gated parallel convolutional swin transformer. Measurement Science and Technology, 36(1), 015104. doi: 10.1088/1361-6501/ad7f77

xxii. Gu, K., Liu, H., Liu, Y., Qiao, J., Zhai, G.,... Zhang, W. (2025). Perceptual Information Fidelity for Quality Estimation of Industrial Images. IEEE Transactions on Circuits and Systems for Video Technology, 35(1), 477-491. doi: 10.1109/TCSVT.2024.3454160

xxiii. Wang, K., Zhu, H., Xu, J., Bai, T., Tian, C., Qian, Y.,... Liu, Y. (2025). Study on the damage mechanism of high-speed turnout switch rails on large ramps. Journal of Central South University, 32(1), 288-303. doi: 10.1007/s11771-025-5858-x

xxiv. Deng, J., Liu, S., Chen, H., Chang, Y., Yu, Y., Ma, W.,... Xie, H. (2025). A Precise Method for Identifying 3D Circles in Freeform Surface Point Clouds. IEEE Transactions on Instrumentation and Measurement. doi: 10.1109/TIM.2025.3547492

xxv. Wang, B., Yang, M., Cao, P., & Liu, Y. (2025). A novel embedded cross framework for high-resolution salient object detection. Applied Intelligence, 55(4), 277. doi: 10.1007/s10489-024-06073-x

xxvi. Li, L., Jin, H., Tu, W., & Zhou, Z. (2024). Study on the minimum safe thickness of water inrush prevention in karst tunnel under the coupling effect of blasting power and water pressure. Tunnelling and Underground Space Technology, 153, 105994. doi: https://doi.org/10.1016/j.tust.2024.105994

xxvii. Cai, H., Wang, Y., Luo, Y., & Mao, K. (2025). A Dual-Channel Collaborative Transformer for continual learning. Applied Soft Computing, 171, 112792. doi: https://doi.org/10.1016/j.asoc.2025.112792

xxviii. Chen, D., Chen, Y., Zhou, Z., Tu, W., & Li, L. (2025). Study on internal rise law of fracture water pressure and progressive fracture mechanism of rock mass under blasting impact. Tunnelling and Underground Space Technology, 161, 106545. doi: https://doi.org/10.1016/j.tust.2025.106545

**Response Reviewer #1:** Thanks for the Reviewer’s comment.

According to the reviewers' suggestions, we have supplemented the introduction with a more detailed analysis related to the research background, especially focusing on the increasing reliance on infrastructure worldwide. Additionally, all the valuable references provided by the reviewers have been cited in the revised manuscript. To address the novelty of our work, we have summarized the key innovative aspects at the end of the introduction section. The specific modifications are as follows:

“The main contributions of this study are summarized as follows:

(1)	A novel multi-scale structural surface damage detection framework is proposed, which integrates the ResNet-50 classification model and the SegFormer segmentation model to effectively handle both coarse- and fine-grained features of structural damage in complex scenes.

(2)	A comprehensive image-based damage quantification pipeline is developed, extracting seven key structural damage parameters from high-resolution images using advanced image processing techniques. These parameters serve as interpretable indicators for safety evaluation.

(3)	An efficient and generalizable safety assessment model based on the RF algorithm is constructed, establishing a nonlinear mapping between damage features and safety levels. Comparative experiments with other machine learning models validate the superiority of the proposed approach.” (On page 5-6, in introduction)

Based on the types and characteristics of structural damage, we propose a multi-scale damage detection algorithm. Structural damages such as cracks, concrete spalling, and exposed reinforcement differ in spatial scale. Cracks are typically slender and difficult to detect due to their subtle image features, whereas spalling and exposed rebar present more distinct pixel-level characteristics, making them relatively easier to identify.

To handle this, our method first uses a classification model to preliminarily segment the damage-related pixel regions in the image domain. These segmented regions are then processed separately by specialized sub-models designed for detecting cracks, spalling, and exposed reinforcement. By filtering out background areas and directing the damage-containing image patches to appropriate sub-models, our approach achieves efficient and accurate detection of various types of structural damage.

**Reviewer #2:** This study utilizes an integrated approach combining machine vision and machine learning to propose a novel method for damage detection and safety assessment of existing structures. The following points have been identified upon reviewing the manuscript:

1. The introduction section should include a discussion of previous studies in the literature that explore alternative damage detection methods, along with their limitations.

2. Please clarify how crack width and length were measured for training the model.

3. The introduction mentions that ultrasonic testing has limitations in damage detection. However, the proposed method primarily detects surface damage and may not provide accurate quantification of in-depth damage, such as the intended depth of cracks or spalling. This limitation should be more clearly addressed.

4. In line 429, the confusion matrix is created for the validation set. Please explain why the testing set was not used instead to evaluate the accuracy of the proposed model.

5. The content in Table 3 is repeated in the text. This redundancy should be removed or revised for conciseness.

6. Please elaborate on how this method can be applied in real-world scenarios. What imaging techniques and equipment are required to perform the detection process?

Overall, the manuscript is well-written, and sufficient information regarding the proposed method is provided. However, the points above should be clarified to enhance the clarity and completeness of the study.

**Response Reviewer #2** Thank you for your thorough review and valuable feedback.

In the revised manuscript, we have updated the introduction to include a more detailed discussion of existing damage detection methods and have clearly articulated the limitations of vision-based approaches. However, as structural surface damage often serves as a visible manifestation of potential underlying issues, it remains a valuable indirect indicator for preliminary safety assessment.

In our experiment, the dataset consisting of 300 samples was divided into training, testing, and validation sets with a ratio of 8:1:1. The training and testing sets were used to optimize model parameters, while the validation set was reserved for evaluating the performance of the safety assessment model. Specifically, we conducted a detailed analysis of the 30 samples in the validation set. It is important to note that neither the testing set nor the validation set was involved in model training.

For clarity and conciseness, we have also refined the description of Table 3. Additionally, we have included a description of the equipment requirements necessary for implementing the proposed method in practical applications. The specific modifications are as follows:

“…To facilitate practical application, the detection process is designed to be compatible with commonly used high-resolution imaging technologies, such as digital single-lens reflex (DSLR) cameras, unmanned aerial vehicle (UAV)-mounted cameras, or mobile device-based imaging systems, depending on the inspection scenario.” (On page 23-24, in Structural safety assessment)

**Reviewer #3:**

1. Model Architecture Design

Strengths: he two-stage approach combining ResNet-50 for classification and SegFormer for segmentation is well-structured. This cascaded framework balances speed and accuracy, which is crucial for real-world deployment. Use of Transformer-based SegFormer leverages long-range dependencies, beneficial for segmenting complex patterns like cracks and spalling.

Suggestions: Consider elaborating on why SegFormer was chosen over other Transformer variants like Swin Transformer or PVT, particularly in the context of computational efficiency or segmentation quality. For ResNet-50, it might be useful to mention any modifications or tuning done specifically for crack detection, e.g., custom loss weights, or early stopping criteria.

2. Loss Function Design

Positive: Using a combined loss function (Cross-Entropy + Dice loss) for segmentation is a solid choice to handle class imbalance in crack vs. background pixels.

Opportunities: Consider reporting the value of alpha (α) used in the combined loss function. It could impact reproducibility and provide insight into loss balancing.

3. Evaluation Metrics

Well done: A good mix of metrics (Accuracy, Precision, Recall, IoU, F1-score) was used for segmentation and classification tasks.

Needs improvement: The segmentation evaluation would benefit from per-class IoU or F1-scores, especially for multi-class damage (e.g., cracks vs. spalling). Consider performing ROC-AUC analysis for binary classification tasks separately to explore classifier behavior under varying thresholds.

4. Dataset and Experimental Setup

Strong points: Data augmentation (rotation, flipping, cropping) improves generalization and is well-explained. The use of real-world and public datasets is appropriate, and splitting into 80:10:10 is standard.

Concerns: Only 200 crack images for segmentation is relatively small. Consider noting how data diversity or transfer learning was handled to counter data sparsity. There’s no mention of image resolution standardization or preprocessing like normalization or color space adjustments. These details are critical in vision models.

5. Feature Engineering for Safety Assessment

The use of seven engineered damage features (e.g., crack length, spalling area, etc.) is commendable and aligns well with domain knowledge. It would help to visualize feature importance from the Random Forest model (e.g., using Gini importance), as this could inform which physical damage metrics are most predictive of structural failure.

6. Machine Learning for Safety Evaluation

The comparison across ML models is informative. RF’s superior performance is expected given its robustness to overfitting and mixed feature types. However, deep learning regressors or hybrid models (e.g., CNN + MLP for end-to-end safety prediction) might offer more scalable alternatives in future work.

7. Technical Reproducibility

While many implementation choices are explained (e.g., optimizer, learning rate), there’s no mention of: Hardware specs (e.g., GPU type, RAM); Training time; Model checkpointing or early stopping criteria. Including these would enhance reproducibility and provide benchmarks for others in the field.

8. Figures and Visualizations

Figures such as segmentation results and skeleton extraction are well-presented. However, figures like Fig. 10 (confusion matrix) would benefit from clearer annotation or color bars for better interpretability.

9. Clarity of Mathematical Formulations

The equations are well-organized and standard in the field. You may consider annotating equations with intuitive explanations or diagrams, especially for loss functions or skeleton length calculations.

10. Future Work

The idea to integrate multi-sensor modalities like IR and ultrasound is forward-thinking. It would be valuable to mention if your model is sensor-agnostic or if retraining is necessary per modality.

**Response Reviewer #3:** We appreciate the time and effort you have put into reviewing our manuscript.

We sincerely thank the reviewer for the thoughtful and detailed feedback. We highly appreciate your recognition of the strengths in our model architecture, loss function design, experimental setup, and overall methodology. Your comments have provided valuable guidance, and we have carefully considered each suggestion during the revision process.

In the revised manuscript, we have elaborated on the rationale behind key model choices—such as the use of SegFormer over other Transformer-based alternatives—emphasizing its balance between segmentation quality and computational efficiency. Details regarding the training of ResNet-50, including early stopping criteria and the training strategy, have been clarified.

To enhance evaluation clarity, we now include per-class segmentation metrics (e.g., F1-scores and IoU) and ROC-AUC analyses where appropriate. We have also added a feature importance visualization for the Random Forest model to highlight the relative contribution of each damage parameter to safety prediction. Furthermore, we expanded the description of preprocessing steps such as resolution normalization and color space adjustment, and supplemented hardware specifications to support reproducibility.

We agree with the reviewer that some aspects, such as the integration of additional Transformer variants, the application of deep learning-based regressors for end-to-end prediction, and the incorporation of multi-sensor modalities, are promising directions. While these were not fully explored in the current study to maintain a focused scope, they are indeed valuable and will be considered in future work.

Once again, we are grateful for your constructive comments, which have significantly improved the clarity, completeness, and rigor of our manuscript. The specific revisions and implementation details can be found in the revised manuscript.

**Reviewer #4:** The authors have done a great job, but they can improve the paper as follows;

1. It will be good if you add to the literature, studies that have performed structural surface damage detection and safety assessment, and their performance metrics

2. All Tables and Figures found in this study are to be referenced similarly in the text. For example, the figure labels have Fig. 1 while Figure 1 is in the text. Make sure to reference all Figures and Tables in the write-up or text similarly. If Fig. 1 is used as the figure label, it must also be referenced in the text as Fig. 1.

3. It will be good to provide a visualization of the proposed models in this study.

4. For Image patch classification model results, a comparative analysis can be performed by using, for example, only the ResNet-50 to run. Also, the Transformer needs to run, and the results from these two can be compared with your proposed model. This will help readers appreciate your work.

5. The accuracy and loss graphs and the confusion matrices of RT, GB, SVM, MLP, and DT must be provided so readers can appreciate the results.

6. Let the novelty of the study come out clearly

**Response Reviewer #4:** Thank you for your professional and insightful review.

We sincerely thank you for the encouraging comments and constructive suggestions. In response, we have carefully reviewed the manuscript and made several adjustments to improve clarity and consistency. Regarding the literature review, we have slightly expanded the introduction to include representative studies related to structural surface damage detection and safety assessment, along with a brief mention of their reported performance metrics, while keeping the discussion focused and concise to avoid disrupting the overall flow. To address the formatting issue, all references to figures and tables in the text have been standardized to match the labels used in the captions (e.g., "Fig. 1").

To intuitively express the effect of each damage assessment model, the confusion matrix of the four groups of comparative experiments is plotted, as shown in Fig 10.

“

|  |  |
| --- | --- |
| (a) GB | (b) SVM |
|  |  |
| (c) MLP | (d) DT |

**Fig 10. Confusion matrix of several comparison models.**”

The proposed damage detection approach aims to take full advantage of both classification and segmentation models. Based on the testing results, the extracted damage features are adequate for supporting the subsequent safety assessment. As such, ablation studies on the classification model were not included in the current study.

Lastly, we have slightly refined the description of our method in the introduction to better highlight the novelty of our multi-model fusion framework for damage detection and safety assessment. The specific revisions and implementation details can be found in the revised manuscript.

**Reviewer #5:**

1. The abstract cannot introduce the purpose and results well.

2. In the introduction, problem statement, and innovation are unclear.

3. The proposed method does not specify what the position of each method is. More explanations are needed.

4. Why is the trend broken according to Figure 3 with 50 iterations?

5. Has the error increased from the 20th iteration onwards?

6. What is the reason for Table 3? What does it prove?

**Response Reviewer #5:** We sincerely appreciate your thoughtful review and the constructive comments you provided.

In the revised version, we have rewritten the abstract to more clearly state the purpose of the study, the methods used, and the key experimental findings. The updated abstract now explicitly highlights the integration of classification and segmentation models, the extracted damage features, the application of the Random Forest-based safety assessment, and the achieved performance metrics.

The introduction has been revised to clearly articulate the limitations of traditional damage detection methods, review prior work on vision-based alternatives, and emphasize the gap in linking surface damage detection with safety assessment. The novelty of our work is now explicitly stated: (1) a multi-scale damage detection approach combining ResNet-50 and SegFormer; (2) extraction of interpretable surface damage features; and (3) integration of these features into a machine learning model for structural safety classification.

ResNet-50 is responsible for coarse-scale image patch classification (e.g., determining whether a region contains damage). SegFormer performs fine-grained pixel-level segmentation to extract detailed features (e.g., crack geometry, spalling area). The Random Forest model then takes the extracted quantitative damage features as input to predict the overall safety level of the structure.

We have re-examined the training/validation curves and clarified in the revised manuscript that although minor fluctuations are observed after the 20th iteration, the overall trend continues to improve or stabilize. These fluctuations are expected in small datasets and do not indicate significant performance degradation. The model exhibited strong predictive performance after 50 training iterations. While it is possible to increase the number of iterations, subsequent tests indicated that further training brought negligible performance gains. As a result, the number of iterations was set to 50 in this study.

Table 3 presents the results of a comparative experiment among several machine learning models (RF, GB, SVM, MLP, and DT) for structural safety classification using the same damage features. It is included to demonstrate the effectiveness and robustness of the proposed Random Forest-based safety assessment approach. The results show that the RF model achieves the highest accuracy, F1-score, and AUC, indicating superior performance in generalizing across different safety categories. We have updated the text to clarify the purpose and implications of this table.
