# Supplementary material for: Structural damage detection and safety assessment method based on machine vision and machine learning
Source: PLoS One. 2026 Feb 2;21(2):e0341653. doi: 10.1371/journal.pone.0341653 (PMC12863553; doi:10.1371/journal.pone.0341653)
Supplement: Attachment — Submitted filename: Response to Reviewers -2.docx [file pone.0341653.s002.docx]

**Responses to the reviewers’ comments**

No.: PONE-D-25-17025R1

Title: Structural Damage Detection and Safety Assessment Method Based on Machine Vision and Machine Learning

Dear editor and reviewers:

Thank you very much for your comments and professional advice. These opinions help to improve academic rigor of our article. The authors have studied reviewer’s comments carefully and have made revision point-by-point response to the reviewer’s comments as provided below. Revisions implemented in accordance are also explained.

Our responses to the reviewers’ comments are given below, and the changed parts are highlighted with red in the revised manuscript. We sincerely hope the revised manuscript would be suitable for publication.

Thank you and best regards.

**Reviewer #2:** The authors have adequately addressed the concerns raised in the previous review round. However, a few minor revisions are still required to improve the clarity of the manuscript:

1. Line 403 (page 24): Please revise the ratio from "8:1:1" to "80:10:10" for improved clarity and consistency, particularly for readers less familiar with part-based notation.

2. Data Availability Statement: The manuscript currently lacks a data availability statement. As per the journal’s guidelines, all supporting data underlying the findings should be made available. Please include a clear data availability statement indicating where and how the data can be accessed, or provide a justification if data cannot be shared.

**Response Reviewer #2** Thank you for your thorough review and valuable feedback.

In response to your suggestion, we have revised the ratio from "8:1:1" to "80:10:10" to enhance clarity and consistency. We have revised the manuscript to include a clear Data Availability Statement in accordance with the journal’s policy. The data used in this study includes both our own experimental data and several publicly available crack detection datasets. Part of the codes used has been uploaded as an attachment. Specifically, the following publicly available datasets were incorporated to expand and validate our results:

**SDNET2018**: A dataset of concrete, brick, and stone surfaces with and without cracks, available at <https://data.mendeley.com/datasets/5y9wdsg2zt/2>

**CRACK500**: A dataset containing annotated crack images for segmentation and classification tasks, available at <https://github.com/cuilimeng/CrackForest-dataset>

**DeepCrack**: A dataset designed for training deep crack detection models, available at <https://github.com/yhlleo/DeepCrack>

CrackForest: A widely used dataset of urban road surface images with corresponding ground truth masks for crack segmentation, available at <https://github.com/cuilimeng/CrackForest-dataset>

Road Crack Detection Dataset (Zou et al.): Includes road surface images with accurate pixel-level crack annotations, available at <https://github.com/ZouCN/Crack_Detection_Dataset>

The specific modifications are as follows:

“…Additionally, some publicly available crack detection datasets (including SDNET2018, CRACK500, DeepCrack, CrackForest, and Road Crack Dataset) were incorporated to expand the datasets [31, 69-73]. After image preprocessing, the dataset was generated as summarized in Table 1. (on page 18)

**Reviewer #3:**

SegFormer Justification (Moderate): While SegFormer is a powerful model, the choice over Swin Transformer or U-Net++ needs better justification, ideally with a benchmark or ablation.

Loss Function Parameter Missing: The combined loss function (Cross-Entropy + Dice) lacks the specific α value. This is important for reproducibility. The dataset (~200 crack images) is modest. A more in-depth discussion on mitigating overfitting (e.g., via transfer learning or synthetic data) is warranted.

Validation vs. Test Set Confusion: Clarify why the validation set was used for final performance reporting instead of an independent test set.

Missing Figure and Table Citations: The manuscript is clear and professionally written, but improvements are needed in the following areas:

Abstract: Needs more concise articulation of the study’s novelty and findings.

Grammar: Phrases like “detection and structural health assessment of structural health” should be corrected for redundancy.

Consistency: Terminology such as “Fig.” vs. “Figure” and spelling variations (e.g., "realise" vs. "realize") should be harmonized.

Formatting: Ensure uniform referencing and cross-referencing of all figures and tables. Inconsistency between "Figure 1" vs. "Fig. 1" should be standardized throughout.

**Response Reviewer #3:** We appreciate the time and effort you have put into reviewing our manuscript.

In response to your comments, we have made the following revisions. Regarding the choice of SegFormer, we have now included a more detailed justification for selecting it over Swin Transformer or U-Net++. The specific modifications are as follows:

“…While Swin Transformer adopts a hierarchical design and window-based self-attention to balance accuracy and efficiency, SegFormer further simplifies the architecture by using lightweight MLP decoders and a pure encoder-decoder structure, making it more suitable for scenarios with limited computational resources. Compared to U-Net++, which relies heavily on CNN-based local operations, SegFormer provides stronger generalization across scales and better global context modeling. This makes it particularly effective for segmenting damage regions with irregular shapes or varying sizes.” (on page 12)

We also agree with your point regarding the missing α value in the combined loss function (Cross-Entropy + Dice). The specific value of $\alpha$ (set to 0.5) has now been included in the manuscript to ensure reproducibility. Additionally, we have expanded the discussion on mitigating overfitting, particularly in the context of the modest dataset (~200 crack images). Specifically, we employed data augmentation techniques (e.g., random flipping, rotation, scaling, and contrast adjustment) and image tiling, where each original image is divided into multiple 112×112 patches, thereby significantly increasing the number of training samples. These strategies help improve model generalization and reduce the risk of overfitting.

We acknowledge that using a separate, independent test set would provide a more rigorous evaluation of generalization performance. However, given the modest dataset size, further splitting into three sets (training/validation/test) would have significantly reduced the number of training samples and potentially compromised model learning. The dataset is currently being expanded, and incorporating an independent test set will be a key consideration in future work.

The abstract has been rewritten to provide a more concise articulation of the study’s novelty and findings. We have ensured consistency in terminology, such as using "Fig." uniformly throughout, and have addressed spelling variations (e.g., "realise" vs. "realize"). Finally, we have checked and standardized the formatting of references and cross-references for figures and tables. The specific modifications are as follows:

“…

Structural damage detection and health assessment are crucial for maintaining infrastructure safety and durability. This study presents a novel multi-scale vision-based framework that combines deep learning and machine learning for accurate and interpretable structural safety evaluation. Specifically, we integrate ResNet-50 and SegFormer models to jointly achieve coarse-level damage classification and fine-grained pixel-level segmentation. Seven key damage parameters are quantitatively extracted from high-resolution images—such as crack length, spalling area, and rebar exposure—and serve as interpretable features for safety assessment. A Random Forest (RF) model is developed to establish a nonlinear mapping from these visual features to structural safety levels. Experimental results demonstrate that the RF-based safety assessment model outperforms other traditional machine learning approaches, achieving an accuracy of 87.0%, F1-score of 0.76, and AUC of 0.83, highlighting its strong generalization and classification capabilities. This work offers a comprehensive and generalizable solution for automated structural damage detection and safety evaluation.” (on page 2)

**Reviewer #4:**

1. The literature studies that have performed structural surface damage detection and safety assessment, and their performance metrics were not presented in the literature.

2. A visualization of the proposed models in this study was not provided, please do provide them.

**Response Reviewer #4:** Thank you for your professional and insightful review.

In response to your comments, we have made the necessary revisions to improve the manuscript. First, we have expanded the literature review to include studies that focus on structural surface damage detection and safety assessment, along with their performance metrics. Additionally, we have included visual representations of the models proposed in this study, as requested. These visualizations provide a clearer understanding of the proposed approach and are now incorporated into the manuscript to enhance the presentation of our work. We hope these revisions address your concerns and further improve the clarity and quality of the manuscript.

The specific modifications are as follows:

“…Several studies have investigated the use of deep learning and machine learning models for structural surface damage detection and safety assessment, reporting promising performance metrics. For example, Zhang et al. developed a CNN-based approach for crack detection on pavement surfaces, achieving classification accuracies exceeding 95% [31]. Cha et al. applied a deep convolutional neural network to detect various types of concrete defects in tunnel linings, reaching a precision of 92.3% and a recall of 90.7% [32]. In terms of safety assessment, Chencho et al. employed a random forest-based approach to quantify element-level structural damage using vibration-derived features, achieving high accuracy and stability in both simulated and experimental scenarios [33]. These studies demonstrate the feasibility of combining surface damage detection with data-driven safety evaluation, although their generalizability across different structural types and image conditions remains a challenge.” (on page 4)

“…The network structure is shown in Table 2.

**Table 2. Network structure table of the classifier.**

| **Layer Index** | **Layer Type** | **Kernel Size / Parameters** | **Output Channels** | **Activation Function** | **Output Dimensions (H×W×C)** |
| --- | --- | --- | --- | --- | --- |
| 1 | Input | – | 3 | – | 112×112×3 |
| 2 | Convolutional | 3×3, stride=1, padding=1 | 64 | ReLU | 112×112×64 |
| 3 | Residual Block ×1 | Two 3×3 convolutions | 64 | ReLU | 112×112×64 |
| 4 | Residual Block ×1 | Two 3×3 convolutions | 64 | ReLU | 112×112×64 |
| 5 | Residual Block ×1 | Two 3×3 convolutions | 64 | ReLU | 112×112×64 |
| 6 | Global Avg Pooling | – | – | – | 1×1×64 |
| 7 | Fully Connected | 64 → 128 | 128 | ReLU | 1×1×128 |
| 8 | Fully Connected | 128 → 2 | 2 | Softmax | 1×1×2 |

” (on page 10)

“…The network structure is shown in Figure 2.

Fig 2. The network structure of SegFormer [45]. (on page 11)
