# Supplementary material for: Structural damage detection and safety assessment method based on machine vision and machine learning
Source: PLoS One. 2026 Feb 2;21(2):e0341653. doi: 10.1371/journal.pone.0341653 (PMC12863553; doi:10.1371/journal.pone.0341653)
Supplement: Attachment — Submitted filename: Response to Reviewers -6.docx [file pone.0341653.s003.docx]

**Responses to the reviewers’ comments**

No.: PONE-D-25-17025R2

Title: Structural Damage Detection and Safety Assessment Method Based on Machine Vision and Machine Learning

Dear editor and reviewers:

Thank you very much for your comments and professional advice. These opinions help to improve academic rigor of our article. The authors have studied reviewer’s comments carefully and have made revision point-by-point response to the reviewer’s comments as provided below. Revisions implemented in accordance are also explained.

Our responses to the reviewers’ comments are given below, and the changed parts are highlighted with red in the revised manuscript. We sincerely hope the revised manuscript would be suitable for publication.

Thank you and best regards.

**Reviewer #6:**

**major questions**

1. The claimed contribution is a "novel multi-scale vision-based framework" combining ResNet-50, SegFormer and Random Forest; in light of many prior works that already use CNN-based classification, transformer-based segmentation and tree-based classifiers for surface damage and safety assessment, what is exactly new in your framework beyond a particular choice of standard models and a specific feature set?

**Response 1:** We thank the reviewer for this comment. The primary contribution of this work is not just the selection of standard models, but the systematic integration of a multi-scale vision pipeline with a physics-informed feature mapping for structural safety. Existing works often treat damage detection as a pure image processing task. Our framework establishes an interpretable bridge by extracting seven specific geometric parameters (aligned with engineering codes) and mapping them to safety levels via RF. This "coarse-to-fine" detection coupled with "interpretable mapping" provides a more transparent decision-making tool for engineers compared to traditional "black-box" end-to-end deep learning models.

2. You pool images and damage samples from various structure types (roads, buildings, bridges, tunnels, hydraulic structures) and both concrete and steel members, but you train a single Random Forest classifier with only seven surface-damage features and three safety levels; how do you justify that the same safety label definition and featureâ€“safety mapping is valid and comparable across such heterogeneous structural systems?

**Response 2**: While the structures are diverse, the visual manifestations of material degradation (e.g., concrete cracking, spalling, and rebar corrosion) follow consistent material-level failure mechanisms. We justified this approach by normalizing safety labels into three "maintenance urgency" levels (S, MD, SD), which are functionally comparable across structural systems. This allows the model to act as a universal preliminary screening tool.

3. The safety assessment dataset consists of only 300 samples, split into 80%/10%/10%; what is the class distribution over S, MD and SD in each split, and is this sample size sufficient to train a 50-tree RF with depth up to 8 without overfitting, especially for the minority classes?

**Response 3**: The 300-sample dataset follows a distribution of S (190), MD (70), and SD (40). To mitigate overfitting on this size, we strictly limited the RF tree depth to 8 and utilized ten-fold cross-validation. RF is inherently robust to small datasets through its bagging mechanism. We have added a class distribution table in Section 4.1.

4. How exactly were the safety labels S, MD and SD generated: which design codes or quantitative criteria were used, were they assigned at element or structure level, how many experts participated, and was inter-rater variability assessed or mitigated?

**Response 4**: Safety labels were assigned at the element level by a panel of five structural experts. They followed the Standard for Maintenance and Strengthening of Concrete Structures and national inspection codes. Inter-rater variability was managed through a consensus-voting process, ensuring a high degree of labeling reliability.

5. The RF model uses only surface-image-based features (crack length, widths, orientation, spalling area, exposed rebar, material type) and does not incorporate load, boundary condition, location of damage within the structural system, or information about hidden deterioration; to what extent can such local surface indicators reliably represent the true structural safety state, and are the conclusions perhaps overstated given these limitations?

**Response 5**: We acknowledge the limitation that surface features cannot fully represent hidden internal states. However, surface damage is often the primary and most visible precursor to structural distress in routine inspections. We have added a discussion acknowledging that this model serves as a rapid preliminary assessment tool, which should be followed by non-destructive testing (NDT) for critical cases.

6. For damage detection, you motivate the use of a cascaded ResNet-50 + SegFormer pipeline but do not provide quantitative comparison with alternative architectures (e.g., U-Net variants, Swin-Transformer-based segmenters, detector-style networks or single-stage segmentation without classification); how do you know that the proposed cascade is actually superior in accuracy“efficiency trade-off?

**Response 6**: SegFormer was selected for its superior global context modeling via self-attention, which is crucial for long, thin crack segmentation. In our supplementary tests, the cascaded ResNet-50 + SegFormer pipeline achieved a better accuracy-efficiency trade-off (reduced false positives by 12% compared to single-stage U-Net) while maintaining high inference speed.

7. The evaluation of the classification and segmentation models is mainly based on training/validation curves and a few qualitative examples; what are the quantitative metrics (accuracy, precision, recall, IoU, F1) on a truly held-out test set, preferably separated by damage type and dataset source, and how do you ensure that there is no data leakage between training, validation and test images?

**Response 7**: We strictly ensured that images from the same structural member were kept within the same split (train, val, or test) to prevent data leakage. Detailed quantitative metrics (Precision, Recall, IoU) for each damage type have been added to the Results section.

8. Equation (5) defines accuracy as (TP + FN) / (TP + TN + FP + FN), which mathematically corresponds to something closer to the complement of specificity rather than accuracy; is this a typographical error and, if so, were all reported accuracy values computed using the correct formula (TP + TN) / (TP + TN + FP + FN)?

**Response 5**: We apologize for this typographical error. The formula for Accuracy in Eq. (5) was meant to be (TP+TN)/(TP+TN+FP+FN). We have confirmed that all reported values were calculated using the correct standard formula.

9. For the crack-geometry quantification you rely on orthogonal skeletonization, region growing and Hough-based orientation estimation, but you do not report any comparison with ground-truth physical measurements (e.g., manual crack lengths and widths measured in the field or on calibration targets); what is the expected measurement error (bias and variance) of your pipeline and how sensitive is the RF safety prediction to these errors?

**Response 9**: To validate quantification accuracy, we compared vision-based measurements with ground-truth manual measurements (using calipers) on 50 samples. The Mean Absolute Error (MAE) for crack width was found to be 0.05 mm, which is well within the acceptable range for field inspections.

10. In the safety assessment results, the Random Forest achieves 87.0% accuracy and perfect recall for class S but shows noticeable confusion between MD and SD, and yet no confidence intervals or statistical tests are reported for the comparison with GB, SVM, MLP and DT; given the relatively small dataset and the practical importance of distinguishing minor from severe damage, how robust and practically acceptable are these performance numbers?

**Response 10:** The confusion between MD and SD reflects the inherent ambiguity in engineering standards where damage thresholds often overlap. We have added 95% confidence intervals to the RF performance results to better demonstrate its statistical robustness compared to other baselines.

**minor questions**

1. Throughout the paper you use both "security" and "safety" to describe the assessment task (e.g., "structural security assessment" vs "structural safety assessment");

**Response 1**: We sincerely apologize for the inconsistent use of terminology. In the context of structural engineering, "safety" is the standard term used to describe the capacity of a structure to resist loads without failure. We have replaced all instances of "security" with "safety" throughout the manuscript, including section titles and attribute labels, to ensure technical accuracy and consistency.

2. Some symbols and abbreviations are introduced without clear definitions the first time they appear (for example, the definition of in Eq. (13) and the use of RF, GB and DT before being expanded in the text);

**Response 2**: We appreciate the reviewer's diligence. We have now provided explicit definitions for all symbols and abbreviations upon their first appearance. Specifically, Ω in Equation (13) is now defined as the domain of the detected spalling region. Abbreviations such as Random Forest (RF), Gradient Boosting (GB), and Decision Tree (DT) are now fully expanded when first mentioned in the text.

3. There are several minor grammatical issues and slightly awkward phrases (for instance, "the learning rate batch size and the number of iterations were set to 0.001, 32, and 200" and "Datasets for security assessment");

**Response 3**: We have conducted a thorough linguistic review of the manuscript to correct grammatical issues and improve the flow of the text. Awkward phrases, such as the description of training hyperparameters and section headings, have been rephrased for better clarity and academic tone.
